# Fumonisin-Induced Disruptions in Sphingolipid Metabolism: Implications for Steroid Hormone Biosynthesis and Hormone Modulation

**DOI:** 10.3390/toxins18070316

**Published:** 2026-07-21

**Authors:** Edward Agyarko, Omeralfaroug Ali, Lucy Ikanya, Attila Zsarnovszky, Melinda Kovács, András Szabó

**Affiliations:** 1 Agrobiotechnology and Precision Breeding for Food Security National Laboratory, Department of Physiology and Animal Health, Institute of Physiology and Nutrition, Hungarian University of Agriculture and Life Sciences, H-7400 Kaposvár, Hungaryszabo.andras@uni-mate.hu (A.S.); 2 Doctoral School of Agriculture and Food Science, Hungarian University of Agriculture and Life Sciences, H-7400 Kaposvár, Hungary; ikanya.lucy.wangari@phd.uni-mate.hu; 3 Department of Farm Animal Nutrition, Institute of Physiology and Nutrition, Hungarian University of Agriculture and Life Sciences, H-7400 Kaposvár, Hungary; 4 HUN-REN-MATE Mycotoxins in the Food Chain Research Group, H-7400 Kaposvár, Hungary

**Keywords:** fumonisins, mycotoxins, sphingolipids, reproductive health, steroid hormones

## Abstract

Fumonisins, a class of mycotoxins produced primarily by *Fusarium* fungi, pose significant health risks to humans and animals through contamination of the food and feed chains. They rank among the most prevalent mycotoxins contaminating maize and maize-derived feeds worldwide, resulting in chronic dietary exposure of both humans and livestock populations across many regions. Their core mechanism of action is the inhibition of ceramide synthases (CerS), which disrupts the essential balance of sphingolipid metabolism by causing an accumulation of free sphingoid bases and a depletion of complex sphingolipids. Both sphingolipids and steroidogenesis are metabolically linked to mitochondrial, membrane and kinase-cascade mechanisms; hence this metabolic disruption may consequently affect steroid hormone biosynthesis, triggering toxicity phenotypes marked by impaired gametogenesis hormonal imbalances, and compromised pregnancy outcomes across mammalian species. Despite the established link between fumonisins and sphingolipid disruption, there is a gap in the literature, as no study to date has integrated sphingolipid disruptions with steroid hormone levels in a dose-dependent manner within reproductive tissues in vivo. This review synthesizes current scientific knowledge across mammalian species to highlight the risks fumonisins pose to reproductive physiology and to identify directions for future research.

## 1. Introduction

Fumonisins, primarily produced by *Fusarium* species of filamentous fungi, are mycotoxins that disrupt the metabolism of sphingolipids in animals by inhibiting all isoforms of ceramide synthase (CerS), the enzymes that are crucial for de novo sphingolipid biosynthesis [1]. A consequence of this inhibition is the accumulation of sphingoid bases, and a parallel depletion of ceramide and its downstream complex sphingolipids. Fumonisins are frequent contaminants of food and animal feed, with corn being the most susceptible commodity, and thus they represent a significant global concern due to their potential to cause substantial economic losses and adverse health effects in both humans and animals [2,3]. In humans, the International Agency for Research on Cancer (IARC) has classified fumonisin $B_{1}$ (FB1) as Group 2B (possibly carcinogenic to humans) [4].

The pervasiveness of fumonisin threat is underscored by a 2024 survey conducted by Dutch State Mines (DSM)-Firmenich, which revealed that 98% of 1275 analyzed finished feed samples across 38 countries tested positive for at least 10 mycotoxins and metabolites [5]. Among these, fumonisins, mostly produced by *Fusarium verticillioides* and *Fusarium proliferatum*, ranked as the second most frequently occurring mycotoxins in completed feed (exceeding 60%), especially in regions characterized by high temperatures and humid climates. Pigs and poultry are among the most exposed livestock species, as fumonisins primarily contaminate maize, a major dietary component for these animals [6,7]. Their widespread occurrence necessitates a thorough mechanistic examination of their primary molecular target, the sphingolipid biosynthetic pathway.

At the cellular level, sphingolipids are defined by a sphingoid base backbone, N-acylated with fatty acids to form ceramide, which is further modified with charged, neutral, phosphorylated, or glycosylated moieties to generate a diverse family of complex sphingolipid molecules [8,9]. Beyond serving as structural membrane components, these lipids and their metabolic derivatives—predominantly ceramide and sphingosine-1-phosphate (So1P)—act as highly potent bioactive signaling mediators [10]. Crucially, sphingolipids coalesce with cholesterol to form specialized, rigid membrane microdomains known as lipid rafts, which serve as essential organizational hubs for receptor clustering and transmembrane signal transduction [11,12]. Because cellular fate is dictated by the precise homeostatic balance between these opposing lipid species—often conceptualized as a rheostat between pro-apoptotic ceramide and pro-survival So1P—any external disruption to the sphingolipid pathway fundamentally compromises cellular integrity [13,14,15,16].

Significant concern regarding the potential impact of fumonisins on steroid hormone biosynthesis stems from their disruption of normal sphingolipid metabolism, given the established roles of sphingolipids in signal transduction, membrane integrity, and the organization of steroidogenic machinery within lipid rafts [17,18]. Steroid hormone biosynthesis represents a critical endocrine function in mammals, responsible for producing a variety of steroid hormones that regulate an extensive array of physiological processes. These hormones are phylogenetically highly conserved mediators of reproduction, metabolism, the development of secondary sexual characteristics, and the maintenance of overall bodily homeostasis [19].

This review aims to bring together the available scientific knowledge concerning fumonisin-induced disruptions of sphingolipid metabolism, analyze their potential effects on steroid hormone biosynthesis pathways, and highlight the resulting implications for their hormone modulator effects on reproductive health across various mammalian species. Within this framework, we define a hormone modulator as a substance that alters circulating or local steroid hormone concentrations by interfering with upstream metabolic pathways [20]; it is distinct from endocrine disruptors whose principal mode of action is a direct interaction with hormonal machinery [21]. This distinction is central to the argument developed in the following sections.

## 2. The Intersection of Steroid Hormone Biosynthesis and Sphingolipid Synthesis

Steroid hormones in mammals—glucocorticoids, mineralocorticoids, progestogens, androgens, and estrogens—are derived from cholesterol, and their production is largely confined to the adrenal cortex, the gonads, the placenta, and, to a lesser extent, the brain and peripheral nervous system [22]. Steroid hormones and sphingolipids are essential regulators of cellular homeostasis, influencing membrane structure, signal transduction, and stress responses. The syntheses of these two lipid classes are not isolated events but are metabolically coupled through shared substrates and regulatory machinery [23].

In the multi-step pathway of steroidogenesis, cholesterol is enzymatically converted into vital glucocorticoids, mineralocorticoids, and sex steroids. The critical rate-limiting step relies on G-protein-coupled receptor signaling cascades that acutely mobilize and shuttle substrate cholesterol across mitochondrial membranes via specialized steroidogenic acute regulatory protein (StAR) and, controversially, the translocator protein (TSPO) [24,25]. It needs to be noted that knockout trials demonstrating intact steroidogenesis in TSPO-deficient mice have cast doubt on TSPO’s crucial involvement in cholesterol transport [26,27]. Once inside the mitochondrion, cholesterol undergoes initial side-chain cleavage by CYP11A1 to form pregnenolone, which serves as the universal precursor for a highly coordinated sequence of tissue-specific cytochrome P 450 (CYP) monooxygenase and 3β-hydroxysteroid dehydrogenase (3β-HSD) enzymes that ultimately dictate systemic reproductive and metabolic capacity [23,28].

**Figure 1:**
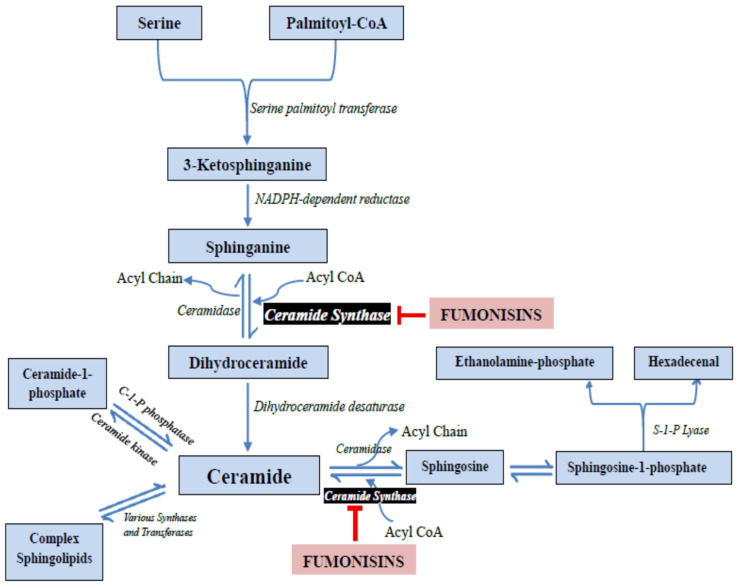
Mechanism of sphingolipid biosynthesis and fumonisin toxicity [31]. (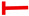 Signifies enzyme inhibition).

The de novo pathway of sphingolipid synthesis (illustrated in Figure 1) begins in the endoplasmic reticulum with the serine palmitoyltransferase (SPT)-catalyzed condensation of L-serine and palmitoyl-CoA to yield 3-keto dihydrosphinganine, which is rapidly reduced to sphinganine (Sa) [16]. This universal intermediate, alongside recycled sphingosine (So) recovered via the cellular salvage pathway, undergoes critical N-acylation by a family of CerS to generate the structural and signaling backbone of ceramide [10]. Because these synthesized sphingolipids and their downstream metabolites (such as So1P) actively serve as secondary messengers that modulate steroidogenic gene expression and direct substrate cholesterol mobilization, any disruption at these enzymatic junctions alters homeostatic steroidogenesis [29,30].

The interplay between steroidogenesis and sphingolipid synthesis is governed by three primary coupling mechanisms that facilitate bidirectional regulation [23,32,33]. This bidirectionality is most clearly demonstrated by a single closed loop traced in the human adrenocortical H295R cell model. Adrenocorticotropic hormone (ACTH), acting through cAMP, rapidly depletes cellular sphingomyelin, ceramide and So while activating sphingosine kinase (SK) and increasing the release of So1P—that is, the trophic signal that drives steroidogenesis is itself a sphingolipid-remodeling signal [34]. The So1P generated in turn promotes cleavage and nuclear translocation of sterol regulatory element-binding protein 1 (SREBP1), which binds the CYP17 promoter and induces its transcription, thereby increasing cortisol output; it also, acutely upregulates StAR, TSPO, SR-BI and LDLR to drive cholesterol delivery to the mitochondrion [35,36]. The loop closes at the level of the master steroidogenic transcription factor itself: steroidogenic factor-1 (SF-1/NR5A1) is normally bound and held inactive by So, and cAMP stimulation measurably decreases the amount of So occupying SF-1, relieving this repression and permitting CYP17 transcription [37]. Genetic evidence confirms that this sphingolipid-to-steroid arm is not a culture artifact: loss-of-function mutations in sphingosine-1-phosphate lyase (SGPL1), the enzyme that irreversibly degrades So1P, cause primary adrenal insufficiency in humans, and Sgpl1−/− mice show disrupted adrenocortical zonation and defective steroidogenic enzyme expression, with acute steroidogenesis directly impaired in patient-derived cells [38]. Together with the reciprocal induction of ceramide-synthesizing enzymes by glucocorticoids, these observations establish a true feedback architecture in which the steroidogenic and sphingolipid pathways continuously reshape one another rather than signaling in a single direction. The three mechanisms described below correspond to a distinct structural and functional levels—mitochondrial/enzymatic, membrane, and kinase-cascade—at which this reciprocal regulation operates.

### 2.1. Regulation of Steroidogenic Enzymes and Mitochondrial Function

Steroid hormone synthesis depends on mitochondrial integrity and cholesterol transport. Ceramide has been shown to influence mitochondrial permeability and regulate StAR activity [23]. This bidirectional relationship is supported experimentally in both directions. Cell-permeable ceramide analogs (C2- and C6-ceramide) reproduce the inhibitory action of tumor necrosis factor-alpha (TNF-α) on StAR protein expression and testosterone biosynthesis in mouse and rat Leydig cells; moreover, intratesticular co-delivery of TNF-α and ceramide directly abrogates StAR expression and steroidogenesis in vivo, identifying ceramide as a downstream transmitter of cytokine signals to the cholesterol-transport machinery [39,40]. Conversely, steroid hormones also feed back onto the sphingolipid arm of the axis: dexamethasone increases hepatic ceramide accumulation by inducing hepatic expression of SPT-2, CerS1 and CerS6, demonstrating that glucocorticoids can actively drive de novo sphingolipid biosynthesis rather than simply respond to it [41].

### 2.2. Membrane Microdomain Modulation

Sphingolipids are essential components of lipid rafts, which serve as platforms for steroid hormone receptors (e.g., estrogen receptor and glucocorticoid receptor). Changes in ceramide and sphingomyelin levels can (a) alter membrane fluidity, (b) modify receptor localization and signaling efficiency, and (c) through sphingolipids localized within membrane lipid rafts acting as extracellular signaling molecules, modulate the hormonal response of neighboring steroidogenic cells [23]. Direct evidence for raft-mediated coupling comes from biochemical fractionation studies. Estrogen receptors co-fractionate with HER-1/HER-2 and flotillin in cholesterol- and sphingolipid-rich caveola-related lipid raft domains isolated from breast cancer cells, placing the extranuclear pool of ER within the same buoyant membrane compartment as its growth-factor signaling partners [42]. Because cholesterol–sphingomyelin-enriched “chol-rafts” and ceramide–sphingomyelin-enriched “cer-rafts” are compositionally and functionally distinct microdomains, a shift in the local ceramide/sphingomyelin ratio provides a plausible mechanism by which sphingolipid metabolism could remodel the raft population available for steroid receptor docking [43].

### 2.3. Signal Transduction Crosstalk

Sphingolipid metabolites such as ceramide and So1P function as second messengers that regulate kinase cascades (e.g., MAPK and PI3K/Akt pathways). These pathways also control steroidogenic enzyme expression (e.g., CYP11A1 and CYP17A1) as well as hormone biosynthesis and secretion [44]. This crosstalk has been demonstrated directly in steroidogenic tissue. In rat Leydig cells, exogenous ceramide and sphingomyelinase suppress hCG-stimulated cAMP production and act at post-cAMP steps of the steroidogenic cascade, indicating that ceramide intercepts the second-messenger cascade at more than one node [45]. In the ovary, So1P generated by luteinizing-hormone- and follicle-stimulating-hormone-induced sphingosine kinase-1 activity modulates gonadotropin-driven steroidogenesis, viability and proliferation in bovine theca and granulosa cells [46,47], and it activates a PLC/PI3K-dependent phosphorylation of the cAMP response element-binding protein (CREB) in human granulosa cells independently of the classical cAMP/PKA route [48]. The convergence of these sphingolipid-responsive kinase cascades on steroidogenic gene expression is further illustrated by evidence that PI3K/Akt signaling directly regulates StAR, CYP11A1 and 3β-HSD transcription in Leydig cells, thereby providing the downstream link between sphingolipid-triggered kinase activation and steroidogenic enzyme output [49].

## 3. Molecular Mechanisms of Fumonisin-Induced Sphingolipid Disruption and Their Biochemical Consequences

The structure of fumonisins resembles that of sphingolipids, and it has been established that the mechanism of toxicity of the most prevalent fumonisin (FB1) is by a strong inhibition of CerSs, the enzymes that N-acylate Sa, So and other sphingoid bases with fatty acyl-CoAs [50]. The results of CerS inhibition by FB1 include reduced formation of dihydroceramides, ceramides, and complex sphingolipids; elevated levels of Sa, So and their 1-phosphates, novel 1-deoxy-sphingoid bases; and alteration in additional lipid metabolites from interrelated pathways [50]. This disruption, illustrated in Figure 1, ultimately leads to changes in overall cellular function.

The existence of six distinct CerS isoforms, each with a preference for acyl chains of specific lengths, implies that fumonisins’ inhibitory effect, although generally directed at CerS, may not be uniform across all ceramide molecular species. Specifically, C18 fatty acyl CoA is attached to the sphingoid base by CerS1; very long fatty acyl CoAs, including C22–C24, are attached by CerS2; C26–C34 acyl CoA is attached by CerS3; C18–C20 fatty acyl CoA is attached by CerS4; and C14–C16 fatty acyl CoA is specifically attached by CerS5 and 6 [51,52]. These CerS isoforms also exhibit a distinct tissue distribution pattern [53]. Consequently, fumonisin exposure produce specific alterations in the profile of ceramide chain lengths within cells and tissues, potentially resulting in diverse downstream consequences depending on which ceramide species are most affected [54]. Indeed, the total ceramide and sphingomyelin content in the lungs and liver has been shown to differ significantly between piglets exposed to FB1 and unexposed controls, signifying an organ-specific adverse effect of FB1 attributable to differential CerS isoform expression [53]. Furthermore, the localization of different enzymes involved in sphingolipid metabolism to specific cellular compartments, such as the endoplasmic reticulum (ER) for de novo synthesis, the Golgi for sphingomyelin synthesis, and lysosomes for the degradation of complex glycosphingolipids, suggests that fumonisins’ primary action in the ER [55] could exert cascading effects on sphingolipid processing and function in other organelles, further complicating the cellular response to these mycotoxins [56]. Furthermore, the well-established roles of sphingolipids in regulating fundamental cellular processes like apoptosis and cell proliferation position them as key mediators of fumonisins’ toxic effects. By disrupting the delicate balance of these lipids fumonisins can shift cellular fate toward either cell death or uncontrolled proliferation, thereby contributing to the various pathologies, possibly including cancer, associated with exposure to these mycotoxins [17].

Fumonisins competitively bind to the active site of CerS; because of the striking resemblance of their aminopentol backbone to Sa and So (the natural substrates of CerS), they effectively prevent the enzyme from catalyzing the N-acylation of Sa (or So) with fatty acyl-CoAs to form dihydroceramide (or ceramide). A further inhibitory effect arises when the aminopentol backbone of FB1 is itself used as a substrate by CerS, leading to the production of an unusual N-acylated analog known as N-palmitoyl-AP1 (PAP1) [57]. Notably, PAP1 has been shown to be an even more potent inhibitor of CerS than FB1 itself, potentially contributing to sustained suppression of enzyme activity following fumonisin exposure [58].

The most prominent biochemical consequence of fumonisin toxicity is the significant accumulation of free sphingoid bases within the cell, primarily Sa, and to a lesser extent, So [59]. This accumulation results from the blockade of the conversion of sphingoid bases to ceramides. As a direct consequence, a characteristic increase in the ratio of sphinganine to sphingosine (Sa/So) is observed in various tissues including the kidney, liver, lung, and heart, as well as in biological fluids such as serum and urine. The increased Sa/So ratio has become a very sensitive biomarker for fumonisin exposure across several mammalian species, serving as an indicator of disrupted sphingolipid metabolism [60]. The proportional rise of Sa and So derives increased synthesis of their phosphorylated derivatives, sphinganine-1-phosphate (Sa1P) and So1P, by the action of SK. SKs remain active during FB1 intoxication and act on the expanded pool of free sphingoid bases as substrate. Thus, CerS inhibition by FB1 triggers elevation of Sa/So and their 1-phosphates, along with the formation of novel 1-deoxy-sphingoid bases. It has been reported that the Sa1P/So1P ratio presents a more apparent marker of fumonisin toxicity compared to the Sa/So ratio in studies involving mice and pigs [61,62]. Conversely, the inhibition of CerS by fumonisins leads to depletion of cellular ceramides, dihydroceramides, and more complex sphingolipids synthesized downstream ceramide in the metabolic pathway. These include important structural and signaling molecules such as sphingomyelin and glycosphingolipids [50].

The disruption of sphingolipid metabolism caused by fumonisins can also trigger compensatory changes in the activities of other enzymes within the pathway. Research has demonstrated that exposure to fumonisins can lead to an increased activity of SPT, the enzyme responsible for catalyzing the initial committed step in the de novo synthesis of sphingolipids [50]. This upregulation of SPT activity likely represents a cellular attempt to overcome the blockage at the CerS step by increasing the production of sphingoid bases. The phosphorylation of Sa and So is also an important compensatory mechanism whereby cells convert cytotoxic, pro-apoptotic sphingoids into signaling molecules.

Fumonisin treatment has further been observed to affect the activities of other enzymes within the sphingolipid metabolic network, such as acid sphingomyelinases (aSMases) and SKs, further contributing to the overall altered sphingolipid profile within cells [54]. Fumonisin exposure can also increase the formation of certain atypical sphingoid bases, such as 1-deoxysphinganine (1-deoxySa). This occurs because SPT can utilize alanine as an alternative substrate to serine in the presence of fumonisins, resulting in the synthesis of these unusual sphingolipids. 1-deoxySa and its subsequent acylated derivatives, known as 1-deoxydihydroceramides, have been shown to possess bioactivity and may contribute to the diverse range of pathological effects associated with fumonisin exposure [54,63]. In a 2021 study using mouse embryonic fibroblasts and an alkyne analog of 1-deoxySa, deoxysphingolipids (deoxySLs) were found to induce autophagosome and lysosome accumulation, indicating an increase in autophagic flux, and thus linking deoxySL pathophysiology to inflammation and the innate immune system [64]. It is interesting that proline-, glutamic acid-, and leucine-rich protein-1 (PELP1), a novel estrogen receptor coactivator, that plays an important role in the genomic and nongenomic actions of estrogen receptors, has been identified in autophagosomes. PELP1, as a co-activator, is upregulated by estrogens and thereby contributes to enhanced autophagy [65]. Disruption of steroidogenesis by fumonisins, therefore, also appears to attenuate the presumably compensatory induction of phagolysosome formation [64]. Autophagosomes play important roles in brain development. Although there is no direct evidence currently linking adverse fumonisin effects to disruption of steroidogenesis, the presence of γ-aminobutyric acid type A receptor associated protein-like 1 (GABARAPL1), a protein responsible for autophagosome formation during brain development, and its persistence in later life in motor neurons [66] allow for the hypothesis that, under FB1 exposure, potential lowered estrogen level (a fundamental trophic hormone in neural development) may lead to developmental deficits in the central nervous system. While intriguing, it is necessary to highlight that this multi-step putative chain linking FB1 exposure to steroidogenesis disruption, PELP1 dysregulation, autophagic impairment and central nervous system developmental deficits, remains hypothetical and unexplored, and should be regarded as a framework for further studies.

The dual action of fumonisins, acting both as competitive inhibitors of CerS and as precursors of even more potent inhibitors, suggests that the effects of fumonisins on sphingolipid metabolism may persist even after initial exposure levels decline, due to the continued presence and activity of their more potent metabolites. These atypical sphingolipids may interfere with other cellular processes or signaling pathways, thereby contributing to the diverse array of health effects observed in association with fumonisin exposure [54,60]. The unique structural properties of 1-deoxy-sphingolipids, specifically the lack of a C1 hydroxyl group (C1-OH), have significant consequences for their behavior within the cell. The missing C1-OH group reduces their ability to form the crucial intra- and intermolecular hydrogen bond networks, that are vital for proper molecular interactions [67]. One consequence is that these atypical sphingolipids do not localize or translocate within cellular compartments as their canonical counterparts do. Studies using fluorescent analogs have shown that 1-deoxy-sphingolipids are not found in the same compartments as normal sphingolipids, indicating that the absence of the C1-OH directly interferes with protein and vesicular transport mechanisms [68]. Also, the N-acylated derivatives of 1-deoxy-sphingolipids are believed to exert a substantial impact on the biophysical properties and overall integrity of cellular membranes, leading to their partial disruption and thereby contributing to cellular damage [69].

## 4. Effects of Fumonisin Exposure on Male and Female Gametogenesis, Hormonal Balance and Pregnancy Outcomes

The role of steroid hormones in the physiological processes of reproduction and development mediated through the hypothalamic–pituitary–gonadal (HPG) axis has been well demonstrated [70,71]. Studies have further shown that sphingolipids can modulate steroid hormone secretion, while steroid hormones can in turn regulate sphingolipid metabolites, indicating the role of sphingolipid metabolites in steroid hormone homeostasis. In this section, the discussion of fumonisin-induced hormonal perturbation in mammals reveals a complex picture, with variations observed depending on the animal species, the sex and developmental stage, the type of cells examined, the steroid hormones being measured, and whether the experiment is conducted in vitro or in vivo [23,72,73]. Table 1 consolidates comparative evidence across these variables providing an overview of fumonisin-induced hormonal effects across species, sexes, and experimental systems. An in vitro study utilizing bovine granulosa cells, which are key players in ovarian steroidogenesis, demonstrated that FB1 alone can enhance the production of estradiol induced by insulin-like growth factor-1 (IGF-1) [74]. In the same study, FB1 was shown to amplify the inhibitory effect of another common mycotoxin, β-zearalenol (β-ZEA), on estradiol production by these cells [74]. However, in a separate in vitro experiment involving bovine granulosa cells, FB1 did not exhibit a significant effect on progesterone production across a range of tested concentrations, although it did cause a slight inhibition of estradiol production at higher doses [75]. The apparent discrepancy between these studies may reflect differences in experimental co-stimulation conditions rather than a species-independent inconsistency: IGF-1/FSH priming and the presence of co-occurring ZEA metabolites are known to modulate whether FB1 exposure stimulates or suppresses granulosa cell steroid output, highlighting a context-dependent mechanism of action [76]. FB1-induced accumulation of free sphingoid bases can initially alter cell signaling kinetics, potentially stabilizing IGF-1R crosstalk with the cAMP pathway to temporarily elevate estradiol synthesis [44]. However, when co-exposed to estrogenic mycotoxins like β-ZEA, the severe depletion of downstream complex sphingolipids likely destabilizes membrane integrity. This disruption impairs cell viability and suppresses crucial steroidogenic enzymes, explaining the transition from promotion to profound inhibition [74]. Furthermore, the variable outcomes observed across higher concentrations in standalone FB1 studies point to a classic biphasic (hormetic) dose–response curve, where low-dose physiological adaptations contrast sharply with high-dose cytotoxicity [75].

It is important to recognize that fumonisins frequently co-occur in contaminated feed with other mycotoxins, such as ZEA and deoxynivalenol (DON), that are, themselves, known to disrupt endocrine functions. These co-occurring mycotoxins can interact differently with the effects of fumonisins on steroidogenesis, potentially producing complex and multifaceted outcomes [74,76,90]. In porcine granulosa cells, FB1 combined with α-ZEA produced an additive increase in progesterone secretion, whereas their combined effect on estradiol diverged from a simple additive pattern [72]. Similarly, in rabbit bucks, subchronic co-exposure to FB1 (5 mg/kg feed) together with DON (1 mg/kg feed) and ZEA (0.25 mg/kg feed) produced a synergistic effect on testosterone production that exceeded what would be expected from fumonisin exposure alone [91], indicating that co-occurring mycotoxins can qualitatively change—not just amplify—fumonisins’ impact on steroid hormone output. These findings underscore that fumonisins’ endocrine impact should be interpreted within the context of realistic mixed-mycotoxin exposure rather than in isolation, though the sphingolipid-mediated mechanism discussed above remains specific to fumonisins themselves.

**Figure 2:**
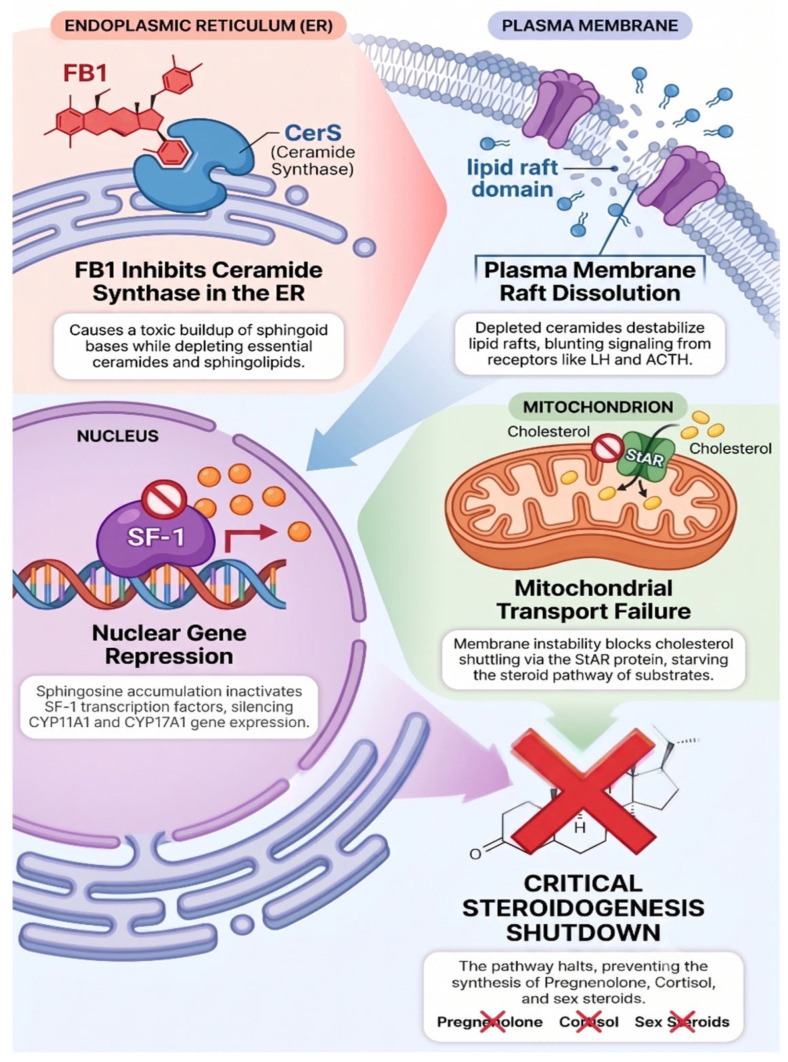
Molecular mechanisms of fumonisin-induced disruption of mammalian steroidogenesis: fumonisin B1 disrupts steroidogenesis by inhibiting ceramide synthesis, leading to cascade effects that include membrane raft destabilization and reduced gene expression for essential enzymes [23,93,94].

Several potential molecular mechanisms have been proposed to explain how fumonisin-induced disruptions in sphingolipid metabolism might ultimately affect the biosynthesis of steroids (Figure 2). One possibility is that the altered sphingolipid profile within steroidogenic cells, particularly the depletion of ceramides, could impair the transport of cholesterol into mitochondria, as already discussed above. This transport, the rate-limiting step of steroidogenesis, is critical, as the initial conversion of cholesterol to pregnenolone by CYP11A1 occurs in the inner mitochondrial membrane [23]. In vivo evidence has reported a marked downregulation of CYP11A1 and CYP11B1 in rats intoxicated with FB1, indicating an FB1-induced disturbance of steroid biosynthesis [92]. Changes in the levels of various sphingolipid metabolites could also directly influence the activity or the expression of key steroidogenic enzymes. This could occur through direct interactions of sphingolipids with these enzymes or indirectly by modulating intracellular signaling pathways (cAMP/PKA, PKC, PI3K/Akt and ERK pathways) that regulate enzyme activity and gene expression. Furthermore, fumonisins’ impact on cellular signaling pathways, which is likely mediated by alterations in sphingolipid metabolism, could indirectly affect the intricate regulatory networks that control the entire process of steroidogenesis [93,94].

In vivo evidence from female rats has demonstrated that dietary exposure to FB1 (10 and 20 mg/kg of feed) reduces serum levels of key gonadotropins essential for regulating reproductive function, such as follicle-stimulating hormone (FSH) and luteinizing hormone (LH). This modulation of hormone levels is associated with decreased fertility rates, shortened gestation lengths, and lower fetal weights in rats [79]. The reduction in gonadotropin levels in rats exposed to fumonisin (at 10 and 20 mg/kg diet) provides a direct link between the mycotoxin and a weakening of HPG axis signaling strength, which is central to regulating reproductive hormone production. This suggests that fumonisins’ impact on steroid hormone biosynthesis may be mediated, at least in part, through this upstream hormonal control. High doses (50 mg/kg b.w./day) of FB1 and FB2 have also been observed to increase the ovary weight index in female rats, potentially indicating an effect on ovarian development, and to reduce the number of primordial, secondary, and tertiary follicles, suggesting an impact on folliculogenesis [80].

In contrast, research in rabbits has found that high doses (20 mg/kg feed) of dietary fumonisins did not exert drastic harmful effects on male reproductive parameters but did slightly stimulate the testicular antioxidant response [82]. In swine, while zearalenone is more prominently linked to reproductive disorders, fumonisins have been associated with abnormal female estrous cycles and reduced litter size [95]. In vitro studies have also indicated that FB1 can negatively affect sperm viability and motility in stallions at concentrations similar to those reported as neurotoxic [88]; however, another study [82] did not find similar effects on spermatocytes in rabbits at 10 and 20 mg/kg feed when assessing membrane and cellular integrity. This discordance underscores the critical difference between direct cellular exposure and systemic pharmacokinetics. In vitro models subject germ cells directly to FB1 without the protective orchestration of the blood–testis barrier (BTB). Because fumonisins are highly polar, their systemic absorption across the gastrointestinal tract is notoriously poor in mono-gastric and lagomorph models; reported oral bioavailability ranges from approximately 3 to 6% in rats, drastically limiting the actual bioavailability of the toxin reaching the testicular parenchyma [82,96].

Maternal exposure to FB1 (at 20 mg/kg b.w./day) during pregnancy has been linked to adverse pregnancy outcomes in mouse models, affecting fetal development [85]. In mice, such exposure has been demonstrated to increase rates of fetal death and decrease fetal weights. Notably, in sensitive mouse strains, FB1 has been shown to induce neural tube defects (NTDs) in developing embryos [76,84,85], even though no NTDs have been recorded in experiments with domestic animals yet [76]. It is currently unclear how FB1 causes NTDs in mice, but it is suspected that it may involve both direct (fetal exposure) and indirect (decreased folate) processes [97]. Similarly, prenatal exposure to high doses of fumonisins in rats has been linked to incomplete ossification (leading to impaired bone development in their offspring), growth delays and even fetal death [98]. In swine, sows that survive acute episodes of pulmonary edema caused by fumonisin exposure during late gestation may subsequently experience abortions. In cattle, the consumption of moldy feed containing various mycotoxins, including fumonisins, has been associated with increased incidences of abortions and infertility [99].

The WHO/IPCS definition of an endocrine disruptor requires demonstration of three criteria: (1) an adverse effect in an intact organism or population, (2) an endocrine mechanism of action, and (3) a plausible causal link between the two. Crucially, the endocrine mechanism must be direct—the substance must interact with hormone receptors, biosynthetic enzymes, or transport proteins as a primary mode of action [21,100,101,102]. Fumonisins’ primary mechanism is CerS inhibition; any hormonal consequence is downstream and indirect, mediated through sphingolipid metabolite imbalance. A hormone modulator, by contrast, is a substance that alters hormonal output through non-receptor-mediated upstream metabolic disruption [20], without fulfilling all three WHO/IPCS criteria simultaneously. The evidence reviewed here demonstrates that fumonisin exposure disrupts steroid hormone production through an indirect, upstream metabolic mechanism—CerS inhibition and the consequent imbalance of bioactive sphingolipid metabolites—rather than through direct interaction with hormone receptors, hormone-synthesizing enzymes, or transport proteins as a primary mode of action. This mechanistic profile aligns with the definition of a hormone modulator rather than with that of a classical endocrine disruptor as defined by WHO/IPCS criteria [21], which require a direct endocrine mechanism causally linked to adverse outcomes in intact organisms [101,103]. Nevertheless, several critical research gaps remain. Firstly, the in vivo quantitative relationship between fumonisin-induced Sa accumulation and measurable suppression of circulating sex steroids has not been established across dose–response gradients in any single mammalian species with sufficient resolution for regulatory risk assessment [75,92,104]. Secondly, the tissue-specific consequences of differential CerS isoform inhibition by FB1 on gonadal versus adrenal steroidogenesis remain uncharacterized [23,104]. Thirdly, the interaction between fumonisins and co-occurring mycotoxins—particularly zearalenone and deoxynivalenol, both of which carry independent endocrine activity—on combined steroidogenic outcomes has not been systematically modeled [74,90].

While dose-dependent sphingolipid disruption by FB1 has been characterized in hepatic and renal tissues of rodents and pigs [92,105,106], and steroid hormone perturbations have been reported independently in gonadal cells [72], no study to date has integrated targeted lipidomic profiling with simultaneous steroid hormone quantification across a dose–response gradient in reproductive tissues in vivo. Therefore, longitudinal in vivo studies employing targeted lipidomics and steroid hormone profiling simultaneously to establish mechanistic dose–response relationships will be a good focus for future research. Another attractive prospect for further research is the application of transcriptomic and proteomic approaches to characterize expression changes of CYP enzymes (some of which are gonad- and adrenal-specific regulated enzymes of the steroidogenic cascade) in gonadal tissue under fumonisin exposure [81,92,107].

## 5. Conclusions

Animals’ exposure to dietary fumonisins has adverse outcomes that may involve various organs both during tissue development and in adulthood. These effects manifest through various signaling and regulatory mechanisms. The plausible relationship between fumonisin exposure and a consequentially disrupted steroid hormone biosynthesis suggests that fumonisins may ultimately exert their ubiquitous effects by decreasing the bioavailability of steroid hormones that play a fundamental role in the regulation of cellular metabolism at all stages of cellular development. Although, to the best of our knowledge, there is no evidence for a direct effect of fumonisins on hormone signaling pathways, and fumonisins therefore cannot be classified as endocrine disruptors, the available evidence from previous studies suggests that fumonisins should be considered indirect hormone modulators.

## Figures and Tables

**Table 1:** Summary of studies analyzing the effects of fumonisins on reproductive hormones and steroidogenic enzyme expression in mammals.

Species, Sex	Experimental Model	Fumonisin, Dose	Hormones Measured	Possible Mechanism/ Affected Enzyme	Key Reproductive Outcome	Reference
Pig, Female	In vitro, porcine granulosa cells	FB1, 10 μM	↑ Progesterone (P4) ↔ Estradiol (E2)	↓ CYP11A1	FB1 ↑ P4 via non-HMG-CoA mechanism; no effect on E2 alone	[72]
Cattle, Female	In vitro, bovine granulosa cells	FB1, 0, 30, 100 ng/mL	↔ P4	Modulates downstream IGF-1 receptor pathways	FB1 increased IGF1-induced E2 production	[74]
Cattle, Female	In vitro, bovine granulosa cells	FB1: 1–30 μM	↓ E2 (slight); ↔ P4	Inhibition of cell proliferation; no effect on CYP11A1 or CYP19A1 mRNA	Significant inhibition of granulosa cell proliferation	[75]
Cow (Holstein), Female	In vivo	FB1 (613.49 µg/kg dry matter (DM)), FB2 (338.06 µg/kg DM)	P4	Modification of physiological post-calving resumption of ovarian activity	↓ Number of corpora lutea; ↓ milk P4 (wk 4–6); ↓ovulation of first dominant follicle	[77]
Goat, Female	In vitro, primary ovarian granulosa cells (GCs)	FB1 (10 μM)	P4, E2	Oxidative stress; mitochondrial dysfunction; G0/G1 arrest; suppression of ERK1/2 signaling; downregulation of steroidogenesis genes	↓GC viability; inhibited steroidogenesis (↓P4 and E2 secretion); apoptosis	[78]
Rat (Wistar), Female	In vivo, dietary exposure (14 days)	FB1: 10 and 20 mg/kg diet	↓ Serum LH; ↓ Serum FSH	Suppression of the hypothalamic–pituitary–ovarian axis	Significant decrease in fertility, gestation length, and fetal weights	[79]
Rat (Wistar Albino), Female	In vivo, prenatal exposure (GD 6–21) to offspring (F1/F2)	FB1: 20 and 50 mg/kg b.w./day	Steroidogenesis genes (inferred E2/P4)	↓ CYP19 mRNA; ↓ ESR2; ↑ LC3 (autophagy in F2)	↑ Ovary weight index (PCOS-like); ↓ primordial follicles (F1/F2); ↓ fertility rate (F1)	[80]
Rat (Wistar) Female	In vivo, early-life exposure (F1 and F2 generations)	FB1 (20 and 50 mg/kg b.w./day)	Not specified	Global DNA hypermethylation; disruption of Pi3k/Akt/mTOR pathway ↑ Dnmt3b (protein), ↑ PI3K, ↓ mTOR	Histopathological damage; degenerating follicles; pyknotic nuclei in GCs; depletion of primordial follicles	[81]
Rabbit, Male	In vivo, dietary exposure (65 days)	FB: 10 and 20 mg/kg diet	Testicular markers	↑ GSH and GSHPx activity; ↓ lipid peroxidation	Negligible effects on testicular weight or sperm parameters	[82]
Rabbit, Male	In vivo, dietary exposure (175 days)	FB1: 5, 7.5, and 10 mg/kg diet	Not reported	Impairment of testicular elements	Delayed puberty; ↓ sperm motility and live count; ↑ sperm abnormalities	[83]
Mouse (LM/Bc), Pregnant Female	In vivo, maternal exposure (ip) on GD 7–8	FB1: 10 mg/kg; HFB1: 2.5–20 mg/kg b.w./day	Not reported	CerS inhibition; ↑ Sa/So ratio; hepatic apoptosis	FB1: 66% neural tube defects (exencephaly); HFB1: No NTDs	[84]
Mouse (LM/Bc), Pregnant Female	In vivo, maternal exposure (ip) GD 7.5–8.5	FB1: 5–20 mg/kg b.w./day	Fetal folate	Disruption of Folbp1 (folate receptor) via depletion of GM1	Dose-dependent increase in neural tube defects (79% at 20 mg/kg)	[85]
Mouse, Male	In vitro, TM4 cells (Sertoli cell line)	FB1 (80 μM)	Not specified (ATP and ROS measured)	Induction of oxidative stress and disruption of glycolysis	Impaired Sertoli cell proliferation; ↑ apoptosis; ↓ ATP production	[86]
Mouse, Male	In vivo	FB1 (5 mg/kg diet)	Keap1-Nrf2 pathway disrupted	Genes in the Nrf2 signal pathway	↑ ROS in Sertoli cells; apoptosis; impaired spermatogenesis	[87]
Horse, Male	In vitro, spermatozoa	FB1: Sub-micromolar and micromolar	Not reported	Sphingoid base accumulation; chromatin instability	↓ Progressive motility; compromised sperm chromatin structure stability	[88]
Human, Female	In vitro, follicular fluid (ff) analysis in IVF patients	FB1 detected in ff (pg/mL range)	E2, P4, anti-Müllerian hormone (AMH)	Low-dose antioxidant role via Nrf2 pathway (speculated)	Positive correlation between ff-FB1 levels and the ratio of oocytes to total follicles	[89]

Abbreviations: CerS, ceramide synthase; E2, estradiol-17β; ER, endoplasmic reticulum; FB1, fumonisin B1; FSH, follicle-stimulating hormone; GSH, glutathione; GSHPx, glutathione peroxidase; HFB1, hydrolyzed fumonisin B1; IGF-1, insulin-like growth factor 1; i.p., intraperitoneal; LH, luteinizing hormone; P4, progesterone; ROS, reactive oxygen species; Sa/So, sphinganine-to-sphingosine ratio.

## Data Availability

No new data were created or analyzed in this study.

## Abbreviations

The following abbreviations are used in this manuscript:

1-deoxySa	1-deoxysphinganine
3β-HSD	3β-Hydroxysteroid dehydrogenase
b.w.	Body weight
β-ZEA	β-zearalenol
cAMP	Cyclic adenosine monophosphate
CerS	Ceramide synthase
CYP	Cytochrome P450
DON	Deoxynivalenol
ER	Endoplasmic reticulum
ERK	Extracellular signal-regulated kinase
FB1	Fumonisin B1
FSH	Follicle-stimulating hormone
HFB1	Hydrolyzed fumonisin B1
HPG	Hypothalamic-pituitary-gonadal
IGF1/IGF-1	Insulin-like growth factor-1
LH	Luteinizing hormone
NTD	Neural tube defect
PI3K/Akt	Phosphoinositide 3-kinase/Protein kinase B
Sa	Sphinganine
Sa1P	Sphinganine-1-phosphate
So1P	Sphingosine-1-phosphate
SK	Sphingosine kinase
So	Sphingosine
SPT	Serine palmitoyltransferase
StAR	Steroidogenic acute regulatory protein
TNF-α	Tumor necrosis factor-alpha
TSPO	Translocator protein
ZEA	Zearalenone

## References

[1] Dellafiora L., Galaverna G., Dall’Asta C. (2018). Mechanisms of Fumonisin B1 Toxicity: A Computational Perspective beyond the Ceramide Synthases Inhibition. Chem. Res. Toxicol..

[2] Li T., Li J., Wang J., Xue K.S., Su X., Qu H., Duan X., Jiang Y. (2024). The occurrence and management of fumonisin contamination across the food production and supply chains. J. Adv. Res..

[3] Voss K.A., Smith G.W., Haschek W.M. (2007). Fumonisins: Toxicokinetics, mechanism of action and toxicity. Anim. Feed Sci. Technol..

[4] International Agency for Research on Cancer Working Group on the Evaluation of Carcinogenic Risks to Humans (2002). Some Traditional Herbal Medicines, Some Mycotoxins, Naphthalene and Styrene.

[5] DSM-Firmenich (2025). DSM-Firmenich World Mycotoxin Survey: The Global Threat–January to December 2024.

[6] Guerre P. (2016). Worldwide Mycotoxins Exposure in Pig and Poultry Feed Formulations. Toxins.

[7] Schrenk D., Bignami M., Bodin L., Chipman J.K., Del Mazo J., Grasl-Kraupp B., Hogstrand C., Leblanc J.C., Nielsen E., Ntzani E. (2022). Assessment of information as regards the toxicity of fumonisins for pigs, poultry and horses. EFSA J..

[8] Merrill A.H., Wang M.D., Park M., Sullards M.C. (2007). (Glyco)sphingolipidology: An amazing challenge and opportunity for systems biology. Trends Biochem. Sci..

[9] Chen Y., Liu Y., Sullards M.C., Merrill A.H. (2010). An introduction to sphingolipid metabolism and analysis by new technologies. NeuroMolecular Med..

[10] Quinville B.M., Deschenes N.M., Ryckman A.E., Walia J.S. (2021). A Comprehensive Review: Sphingolipid Metabolism and Implications of Disruption in Sphingolipid Homeostasis. Int. J. Mol. Sci..

[11] Gault C.R., Obeid L.M., Hannun Y.A. (2010). An overview of sphingolipid metabolism: From synthesis to breakdown. Adv. Exp. Med. Biol..

[12] Wright N.J. (2016). A Beginners Guide to the Metabolism, Functions and Pharmacological Potential of Sphingolipids. J. Pharmacol. Pharm. Pharmacovigil..

[13] Bellini L., Campana M., Mahfouz R., Carlier A., Véret J., Magnan C., Hajduch E., Le Stunff H. (2015). Targeting sphingolipid metabolism in the treatment of obesity/type 2 diabetes. Expert Opin. Ther. Targets.

[14] Hannun Y.A., Obeid L.M. (2008). Principles of bioactive lipid signalling: Lessons from sphingolipids. Nat. Rev. Mol. Cell Biol..

[15] Mencarelli C., Martinez-Martinez P. (2013). Ceramide function in the brain: When a slight tilt is enough. Cell. Mol. Life Sci..

[16] Young S., Mina J., Denny P., Smith T. (2012). Sphingolipid and Ceramide Homeostasis: Potential Therapeutic Targets. Biochem. Res. Int..

[17] Riley R.T., Enongene E., Voss K.A., Norred W.P., Meredith F.I., Sharma R.P., Spitsbergen J., Williams D.E., Carlson D.B., Merrill A.H. (2001). Sphingolipid perturbations as mechanisms for fumonisin carcinogenesis. Env. Health Perspect..

[18] Khoury D.E., Fayjaloun S., Nassar M., Sahakian J., Aad P.Y. (2019). Updates on the Effect of Mycotoxins on Male Reproductive Efficiency in Mammals. Toxins.

[19] Sanderson J.T. (2006). The Steroid Hormone Biosynthesis Pathway as a Target for Endocrine-Disrupting Chemicals. Toxicol. Sci..

[20] Guarnotta V., Amodei R., Frasca F., Aversa A., Giordano C. (2022). Impact of Chemical Endocrine Disruptors and Hormone Modulators on the Endocrine System. Int. J. Mol. Sci..

[21] International Programme on Chemical Safety (2002). Global Assessment on the State of the Science of Endocrine Disruptors.

[22] Holst J.P., Soldin O.P., Guo T., Soldin S.J. (2004). Steroid hormones: Relevance and measurement in the clinical laboratory. Clin. Lab. Med..

[23] Lucki N., Sewer M. (2010). The Interplay Between Bioactive Sphingolipids and Steroid Hormones. Steroids.

[24] Baranowski E.S., Arlt W., Idkowiak J. (2018). Monogenic Disorders of Adrenal Steroidogenesis. Horm. Res. Paediatr..

[25] Batth R., Nicolle C., Cuciurean I.S., Simonsen H.T. (2020). Biosynthesis and Industrial Production of Androsteroids. Plants.

[26] Banati R.B., Middleton R.J., Chan R., Hatty C.R., Kam W.W.-Y., Quin C., Graeber M.B., Parmar A., Zahra D., Callaghan P. (2014). Positron emission tomography and functional characterization of a complete PBR/TSPO knockout. Nat. Commun..

[27] Liere P., Liu G.-J., Pianos A., Middleton R.J., Banati R.B., Akwa Y. (2023). The Comprehensive Steroidome in Complete TSPO/PBR Knockout Mice under Basal Conditions. Int. J. Mol. Sci..

[28] Vinson G.P. (2016). Functional Zonation of the Adult Mammalian Adrenal Cortex. Front. Neurosci..

[29] Pralhada Rao R., Vaidyanathan N., Rengasamy M., Mammen Oommen A., Somaiya N., Jagannath M.R. (2013). Sphingolipid metabolic pathway: An overview of major roles played in human diseases. J. Lipids.

[30] Brice S.E., Cowart L.A. (2011). Sphingolipid metabolism and analysis in metabolic disease. Adv. Exp. Med. Biol..

[31] Ajithkumar K., Renuka M., Savitha A.S., Yenjereappa S.T. (2022). Fumonisin: A Potential Mycotoxin Produced by Fusarium verticellioides and it’s Impact on Human and Animal Health. Adv. Agric. Technol. Plant Sci..

[32] Cai K., Lucki N.C., Sewer M.B. (2014). Silencing diacylglycerol kinase-theta expression reduces steroid hormone biosynthesis and cholesterol metabolism in human adrenocortical cells. Biochim. Biophys. Acta.

[33] Fyrst H., Saba J.D. (2010). An update on sphingosine-1-phosphate and other sphingolipid mediators. Nat. Chem. Biol..

[34] Ozbay T., Rowan A., Leon A., Patel P., Sewer M.B. (2006). Cyclic adenosine 5′-monophosphate-dependent sphingosine-1-phosphate biosynthesis induces human CYP17 gene transcription by activating cleavage of sterol regulatory element binding protein 1. Endocrinology.

[35] Lucki N.C., Li D., Sewer M.B. (2012). Sphingosine-1-phosphate rapidly increases cortisol biosynthesis and the expression of genes involved in cholesterol uptake and transport in H295R adrenocortical cells. Mol. Cell. Endocrinol..

[36] Urs A.N., Dammer E., Sewer M.B. (2006). Sphingosine Regulates the Transcription of CYP17 by Binding to Steroidogenic Factor-1. Endocrinology.

[37] Prasad R., Hadjidemetriou I., Maharaj A., Meimaridou E., Buonocore F., Saleem M., Hurcombe J., Bierzynska A., Barbagelata E., Bergadá I. (2017). Sphingosine-1-phosphate lyase mutations cause primary adrenal insufficiency and steroid-resistant nephrotic syndrome. J. Clin. Investig..

[38] Maharaj A., Williams J., Bradshaw T., Güran T., Braslavsky D., Casas J., Chan L.F., Metherell L.A., Prasad R. (2020). Sphingosine-1-phosphate lyase (SGPL1) deficiency is associated with mitochondrial dysfunction. J. Steroid Biochem. Mol. Biol..

[39] Budnik L.T., Jähner D., Mukhopadhyay A.K. (1999). Inhibitory effects of TNF alpha on mouse tumor Leydig cells: Possible role of ceramide in the mechanism of action. Mol. Cell. Endocrinol..

[40] Morales V., Santana P., Díaz R., Tabraue C., Gallardo G., López Blanco F., Hernández I., Fanjul L.F., Ruiz de Galarreta C.M. (2003). Intratesticular delivery of tumor necrosis factor-alpha and ceramide directly abrogates steroidogenic acute regulatory protein expression and Leydig cell steroidogenesis in adult rats. Endocrinology.

[41] Holland W.L., Brozinick J.T., Wang L.-P., Hawkins E.D., Sargent K.M., Liu Y., Narra K., Hoehn K.L., Knotts T.A., Siesky A. (2007). Inhibition of Ceramide Synthesis Ameliorates Glucocorticoid-, Saturated-Fat-, and Obesity-Induced Insulin Resistance. Cell Metab..

[42] Márquez D.C., Chen H.W., Curran E.M., Welshons W.V., Pietras R.J. (2006). Estrogen receptors in membrane lipid rafts and signal transduction in breast cancer. Mol. Cell. Endocrinol..

[43] Patel H.H., Insel P.A. (2009). Lipid rafts and caveolae and their role in compartmentation of redox signaling. Antioxid. Redox Signal..

[44] Wang D., Tang Y., Wang Z. (2023). Role of sphingolipid metabolites in the homeostasis of steroid hormones and the maintenance of testicular functions. Front. Endocrinol..

[45] Meroni S.B., Pellizzari E.H., Cánepa D.F., Cigorraga S.B. (2000). Possible involvement of ceramide in the regulation of rat Leydig cell function. J. Steroid Biochem. Mol. Biol..

[46] González-Aretia D., Hernández-Coronado C.G., Guzmán A., Medina-Moctezuma Z.B., Gutiérrez C.G., Rosales-Torres A.M. (2024). Sphingosine-1-phosphate mediates FSH-induced cell viability but not steroidogenesis in bovine granulosa cells. Theriogenology.

[47] Medina-Moctezuma Z.B., Hernández-Coronado C.G., Marín-López L., Guzmán A., González-Aretia D., Gutiérrez C.G., Rosales-Torres A.M. (2023). Sphingosine-1-phosphate regulation of luteinising hormone-induced steroidogenesis and proliferation of bovine theca cells in vitro. Reprod. Fertil. Dev..

[48] Paradiso E., Lazzaretti C., Sperduti S., Antoniani F., Fornari G., Brigante G., Di Rocco G., Tagliavini S., Trenti T., Morini D. (2021). Sphingosine-1 phosphate induces cAMP/PKA-independent phosphorylation of the cAMP response element-binding protein (CREB) in granulosa cells. Mol. Cell. Endocrinol..

[49] Xu H., Pu J., Teng Y., Zhu Q., Guo L., Zhao J., Ding H., Fang Y., Ma X., Liu H. (2023). Melatonin Inhibits Testosterone Synthesis in Rooster Leydig Cells by Targeting CXCL14 through miR-7481-3p. Int. J. Mol. Sci..

[50] Riley R.T., Merrill A.H. (2019). Ceramide synthase inhibition by fumonisins: A perfect storm of perturbed sphingolipid metabolism, signaling, and disease. J. Lipid Res..

[51] Raichur S. (2020). Ceramide Synthases Are Attractive Drug Targets for Treating Metabolic Diseases. Front. Endocrinol..

[52] Mullen T.D., Hannun Y.A., Obeid L.M. (2012). Ceramide synthases at the centre of sphingolipid metabolism and biology. Biochem. J..

[53] Loiseau N., Polizzi A., Dupuy A., Therville N., Rakotonirainy M., Loy J., Viadere J.L., Cossalter A.M., Bailly J.D., Puel O. (2015). New insights into the organ-specific adverse effects of fumonisin B1: Comparison between lung and liver. Arch. Toxicol..

[54] He Q., Suzuki H., Sharma N., Sharma R.P. (2006). Ceramide Synthase Inhibition by Fumonisin B1 Treatment Activates Sphingolipid-Metabolizing Systems in Mouse Liver. Toxicol. Sci..

[55] Pascoa T.C., Pike A.C.W., Tautermann C.S., Chi G., Traub M., Quigley A., Chalk R., Štefanić S., Thamm S., Pautsch A. (2025). Structural basis of the mechanism and inhibition of a human ceramide synthase. Nat. Struct. Mol. Biol..

[56] Canals D., Clarke C.J. (2022). Compartmentalization of Sphingolipid metabolism: Implications for signaling and therapy. Pharmacol. Ther..

[57] Humpf H.U., Schmelz E.M., Meredith F.I., Vesper H., Vales T.R., Wang E., Menaldino D.S., Liotta D.C., Merrill A.H. (1998). Acylation of naturally occurring and synthetic 1-deoxysphinganines by ceramide synthase. Formation of N-palmitoyl-aminopentol produces a toxic metabolite of hydrolyzed fumonisin, AP1, and a new category of ceramide synthase inhibitor. J. Biol. Chem..

[58] Seiferlein M., Humpf H.U., Voss K.A., Sullards M.C., Allegood J.C., Wang E., Merrill A.H. (2007). Hydrolyzed fumonisins HFB1 and HFB2 are acylated in vitro and in vivo by ceramide synthase to form cytotoxic N-acyl-metabolites. Mol. Nutr. Food Res..

[59] Wang E., Ross P.F., Wilson T.M., Riley R.T., Merrill A.H. (1992). Increases in serum sphingosine and sphinganine and decreases in complex sphingolipids in ponies given feed containing fumonisins, mycotoxins produced by Fusarium moniliforme. J. Nutr..

[60] Voss K.A., Riley R.T. (2013). Fumonisin Toxicity and Mechanism of Action: Overview and Current Perspectives. Food Saf..

[61] Kim D.H., Yoo H.S., Lee Y.M., Kie J.H., Jang S., Oh S. (2006). Elevation of sphinganine 1-phosphate as a predictive biomarker for fumonisin exposure and toxicity in mice. J. Toxicol. Environ. Health A.

[62] Lassallette E., Pierron A., Tardieu D., Reymondaud S., Gallissot M., Rodriguez M.A., Collén P.N., Roy O., Guerre P. (2025). Biomarkers of Fumonisin Exposure in Pigs Fed the Maximum Recommended Level in Europe. Toxins.

[63] Zitomer N.C., Mitchell T., Voss K.A., Bondy G.S., Pruett S.T., Garnier-Amblard E.C., Liebeskind L.S., Park H., Wang E., Sullards M.C. (2009). Ceramide synthase inhibition by fumonisin B1 causes accumulation of 1-deoxysphinganine: A novel category of bioactive 1-deoxysphingoid bases and 1-deoxydihydroceramides biosynthesized by mammalian cell lines and animals. J. Biol. Chem..

[64] Lauterbach M.A., Saavedra V., Mangan M.S.J., Penno A., Thiele C., Latz E., Kuerschner L. (2021). 1-Deoxysphingolipids cause autophagosome and lysosome accumulation and trigger NLRP3 inflammasome activation. Autophagy.

[65] Ohshiro K., Rayala S.K., Kondo S., Gaur A., Vadlamudi R.K., El-Naggar A.K., Kumar R. (2007). Identifying the estrogen receptor coactivator PELP1 in autophagosomes. Cancer Res..

[66] Le Grand J.N., Bon K., Fraichard A., Zhang J., Jouvenot M., Risold P.Y., Boyer-Guittaut M., Delage-Mourroux R. (2013). Specific distribution of the autophagic protein GABARAPL1/GEC1 in the developing and adult mouse brain and identification of neuronal populations expressing GABARAPL1/GEC1. PLoS ONE.

[67] Lone M.A., Santos T., Alecu I., Silva L.C., Hornemann T. (2019). 1-Deoxysphingolipids. Biochim. Biophys. Acta Mol. Cell Biol. Lipids.

[68] Tsai Y.T., Lipp N.F., Seidel O., Varma R., Laguerre A., Solorio-Kirpichyan K., Wong A., Brea R.J., McGregor G.H., Cordes T. (2025). 1-Deoxysphingolipids dysregulate membrane properties and cargo trafficking in the early secretory pathway. bioRxiv.

[69] Jiménez-Rojo N., Sot J., Busto J.V., Shaw W.A., Duan J., Merrill A.H., Alonso A., Goñi F.M. (2014). Biophysical properties of novel 1-deoxy-(dihydro)ceramides occurring in mammalian cells. Biophys. J..

[70] Tyree M.F., Stenhouse C. (2025). Exogenous progesterone supplementation: A strategy to enhance conceptus development in sheep and pigs?. Reprod. Fertil..

[71] Spencer T.E., Johnson G.A., Bazer F.W., Burghardt R.C., Palmarini M. (2007). Pregnancy recognition and conceptus implantation in domestic ruminants: Roles of progesterone, interferons and endogenous retroviruses. Reprod. Fertil. Dev..

[72] Cortinovis C., Caloni F., Schreiber N.B., Spicer L.J. (2014). Effects of fumonisin B_1_ alone and combined with deoxynivalenol or zearalenone on porcine granulosa cell proliferation and steroid production. Theriogenology.

[73] Rheeder J.P., Marasas W.F., Vismer H.F. (2002). Production of fumonisin analogs by Fusarium species. Appl. Environ. Microbiol..

[74] Albonico M., Schütz L.F., Caloni F., Cortinovis C., Spicer L.J. (2016). Toxicological effects of fumonisin B1 alone and in combination with other fusariotoxins on bovine granulosa cells. Toxicon.

[75] Albonico M., Schutz L.F., Caloni F., Cortinovis C., Spicer L.J. (2017). In vitro effects of the *Fusarium mycotoxins* fumonisin B_1_ and beauvericin on bovine granulosa cell proliferation and steroid production. Toxicon.

[76] Cortinovis C., Pizzo F., Spicer L.J., Caloni F. (2013). *Fusarium mycotoxins*: Effects on reproductive function in domestic animals—A review. Theriogenology.

[77] Catellani A., Mossa F., Gabai G., D’Hallewin J.S.K., Trevisi E., Faas J., Artavia I., Labudova S., Piccioli-Cappelli F., Minuti A. (2025). Efficacy of a mycotoxin-deactivating product to reduce the impact of Fusarium mycotoxin-contaminated rations in dairy cows during early lactation. J. Dairy Sci..

[78] Li Z., Huang J., Xiao M., Zhou B., Liu H., Chen J., Ruan Y. (2026). Fumonisin B1-induced oxidative stress-mediated damage in goat ovarian granulosa cells and the protective role of rutin. Anim. Reprod. Sci..

[79] Gbore F., Owolawi T., Erhunwunsee M., Akele O., Gabriel-Ajobiewe R. (2012). Evaluation of the Reproductive Toxicity of Dietary Fumonisin B 1 in Rats. Jordan J. Biol. Sci..

[80] Alhelaisi A., Alrezaki A., Nahdi S., Aldahmash W., Alwasel S., Harrath A.H. (2023). Early-Life Exposure to the Mycotoxin Fumonisin B1 and Developmental Programming of the Ovary of the Offspring: The Possible Role of Autophagy in Fertility Recovery. Toxics.

[81] Alhelaisi A., Nahdi S., Alhazmi A., Alwasel S., Harrath A.H. (2025). Dynamic Changes of Selected Signaling Molecules in Ovaries Following Early-Life Exposure to Fumonisin B1 in Wistar Rats in Association With DNA Methylation. Physiol. Res..

[82] Szabó A., Nagy S., Ali O., Zsolt G., Mezes M., Balogh K., Bartók T., Horváth L., Mouhanna A., Kovacs M. (2021). A 65-Day Fumonisin B Exposure at High Dietary Levels Has Negligible Effects on the Testicular and Spermatological Parameters of Adult Rabbit Bucks. Toxins.

[83] Ewuola E.O., Egbunike G.N. (2010). Effects of dietary fumonisin B1 on the onset of puberty, semen quality, fertility rates and testicular morphology in male rabbits. Reproduction.

[84] Voss K.A., Riley R.T., Snook M.E., Gelineau-van Waes J. (2009). Reproductive and sphingolipid metabolic effects of fumonisin B1 and its alkaline hydrolysis product in LM/Bc mice: Hydrolyzed fumonisin B1 did not cause neural tube defects. Toxicol. Sci..

[85] Gelineau-van Waes J., Starr L., Maddox J., Aleman F., Voss K.A., Wilberding J., Riley R.T. (2005). Maternal fumonisin exposure and risk for neural tube defects: Mechanisms in an in vivo mouse model. Birth Defects Res. Part A Clin. Mol. Teratol..

[86] Ma J., Huang R., Zhang H., Liu D., Dong X., Xiong Y., Xiong X., Lan D., Fu W., He H. (2024). The Protective Effect of Quercetin against the Cytotoxicity Induced by Fumonisin B1 in Sertoli Cells. Int. J. Mol. Sci..

[87] Ouyang H., Zhu H., Li J., Chen L., Zhang R., Fu Q., Li X., Cao C. (2022). Fumonisin B1 promotes germ cells apoptosis associated with oxidative stress-related Nrf2 signaling in mice testes. Chem.-Biol. Interact..

[88] Minervini F., Lacalandra G.M., Filannino A., Garbetta A., Nicassio M., Dell’Aquila M.E., Visconti A. (2010). Toxic effects induced by mycotoxin fumonisin B1 on equine spermatozoa: Assessment of viability, sperm chromatin structure stability, ROS production and motility. Toxicol. Vitro.

[89] Szentirmay A., Molnár Z., Plank P., Mézes M., Sajgó A., Martonos A., Buzder T., Sipos M., Hruby L., Szőke Z. (2024). The Potential Influence of the Presence of Mycotoxins in Human Follicular Fluid on Reproductive Outcomes. Toxins.

[90] Demaegdt H., Daminet B., Evrard A., Scippo M.-L., Muller M., Pussemier L., Callebaut A., Vandermeiren K. (2016). Endocrine activity of mycotoxins and mycotoxin mixtures. Food Chem. Toxicol..

[91] Fodor J. (2015). Individual and Combined Effects of Subchronic Exposure of Three Fusarium Toxins (Fumonisin B, Deoxynivalenol and Zearalenone) in Rabbit Bucks. J. Clin. Toxicol..

[92] Szabó A., Péter M., Balogh G., Török Z., Kóta Z., Vígh L., Ali O., Timár B., Kövér G., Nagy T. (2025). Liver Lipidomics, Histology, Transcriptomics, and Clinical Chemistry of Rats Intraperitoneally Treated with Fumonisin B1 for 5 days. J. Agric. Food Chem..

[93] Lucki N.C., Sewer M.B. (2008). Multiple roles for sphingolipids in steroid hormone biosynthesis. Subcell. Biochem..

[94] Jiang X.C., Li Z. (2022). Sphingolipids and Cholesterol. Adv. Exp. Med. Biol..

[95] Fromme H., Gareis M., Völkel W., Gottschalk C. (2016). Overall internal exposure to mycotoxins and their occurrence in occupational and residential settings—An overview. Int. J. Hyg. Environ. Health.

[96] Knutsen H.K., Alexander J., Barregård L., Bignami M., Brüschweiler B., Ceccatelli S., Cottrill B., Dinovi M., Edler L., Grasl-Kraupp B. (2018). Risks for animal health related to the presence of fumonisins, their modified forms and hidden forms in feed. EFSA J..

[97] Stevens V.L., Tang J. (1997). Fumonisin B1-induced Sphingolipid Depletion Inhibits Vitamin Uptake via the Glycosylphosphatidylinositol-anchored Folate Receptor. J. Biol. Chem..

[98] Tomaszewska E., Rudyk H., Muszyński S., Hułas-Stasiak M., Leszczyński N., Mielnik-Błaszczak M., Donaldson J., Dobrowolski P. (2023). Prenatal Fumonisin Exposure Impairs Bone Development via Disturbances in the OC/Leptin and RANKL/RANK/OPG Systems in Weaned Rat Offspring. Int. J. Mol. Sci..

[99] Muñoz-Solano B., Lizarraga Pérez E., González-Peñas E. (2024). Monitoring Mycotoxin Exposure in Food-Producing Animals (Cattle, Pig, Poultry, and Sheep). Toxins.

[100] Andersson N., Arena M., Auteri D., Barmaz S., Grignard E., Kienzler A., Lepper P., Lostia A.M., Munn S., European Chemical Agency (ECHA) and European Food Safety Authority (EFSA) with the technical support of the Joint Research Centre (JRC) (2018). Guidance for the identification of endocrine disruptors in the context of Regulations (EU) No 528/2012 and (EC) No 1107/2009. EFSA J..

[101] Zoeller R.T., Brown T.R., Doan L.L., Gore A.C., Skakkebaek N.E., Soto A.M., Woodruff T.J., Vom Saal F.S. (2012). Endocrine-disrupting chemicals and public health protection: A statement of principles from The Endocrine Society. Endocrinology.

[102] Zoeller R.T., Bergman Å., Becher G., Bjerregaard P., Bornman R., Brandt I., Iguchi T., Jobling S., Kidd K.A., Kortenkamp A. (2014). A path forward in the debate over health impacts of endocrine disrupting chemicals. Environ. Health.

[103] Diamanti-Kandarakis E., Bourguignon J.P., Giudice L.C., Hauser R., Prins G.S., Soto A.M., Zoeller R.T., Gore A.C. (2009). Endocrine-disrupting chemicals: An Endocrine Society scientific statement. Endocr. Rev..

[104] Yamazoe Y., Koyama N., Kumagai S. (2017). Possible Role of Phosphatidylcholine and Sphingomyelin on Fumonisin B1-mediated Toxicity. Food Saf..

[105] Direito G.M., Almeida A.P., Aquino S., Alves Dos Reis T., Rodrigues Pozzi C., Corrêa B. (2009). Evaluation of sphingolipids in Wistar rats treated to prolonged and single oral doses of fumonisin b_1_. Int. J. Mol. Sci..

[106] Szabó A., Szabó-Fodor J., Kachlek M., Mézes M., Balogh K., Glávits R., Ali O., Zeebone Y.Y., Kovács M. (2018). Dose and Exposure Time-Dependent Renal and Hepatic Effects of Intraperitoneally Administered Fumonisin B_1_ in Rats. Toxins.

[107] Gamiet N., Deepnarain N., Abel S., Burger H.-M., Mayer E., Lilly M. (2025). Comparative proteomic analysis indicates differential responses to fumonisin B1 (FB1) and hydrolysed fumonisin B1 (HFB1) in IPEC-J2 porcine epithelial cells in vitro. Mycotoxin Res..

